# Author Correction: KDM6B*/Pdk1* glycolytic pathway-driven ZEB2 lactylation promotes cellular cementum formation

**DOI:** 10.1038/s41368-026-00453-4

**Published:** 2026-07-15

**Authors:** Zhengkun Yang, Huiyi Wang, Junhong Xiao, Qiudong Yang, Jiahui Sun, Heyu Liu, Zhendong Huang, Li Ma, Xin Huang, Chuan Wang, Xiaoxuan Wang, Zhengguo Cao

**Affiliations:** 1 State Key Laboratory of Oral & Maxillofacial Reconstruction and Regeneration, Key Laboratory of Oral Biomedicine, Ministry of Education, Hubei Key Laboratory of Stomatology, School & Hospital of Stomatology, Wuhan University, Wuhan, China; 2 Department of Periodontology, School & Hospital of Stomatology, Wuhan University, Wuhan, China; 3 Qingdao Stomatological Hospital Affiliated to Qingdao University, Qingdao, China

**Keywords:** Mechanisms of disease, Extracellular signalling molecules

Correction to: *International Journal of Oral Science* 10.1038/s41368-025-00420-5, published online 03 March 2026

Following the publication of original article^1^, it is reported that an incorrect version of Fig. 1e, 1f, and Fig. 5f are used in the published version. During the initial submission and revision, correct figures were submitted; However, after the article was accepted, the authors mistakenly provided an internal pre-submission version of the figures for production, leading to discrepancies. Regrettably, this error was not detected during the proofreading stage by authors.

Both the originally intended figures and the erroneously published figures represent the same indicators derived from samples under identical conditions, and the conclusions of the paper remain unchanged.

Figure 1 has been corrected from:

**Figure:**
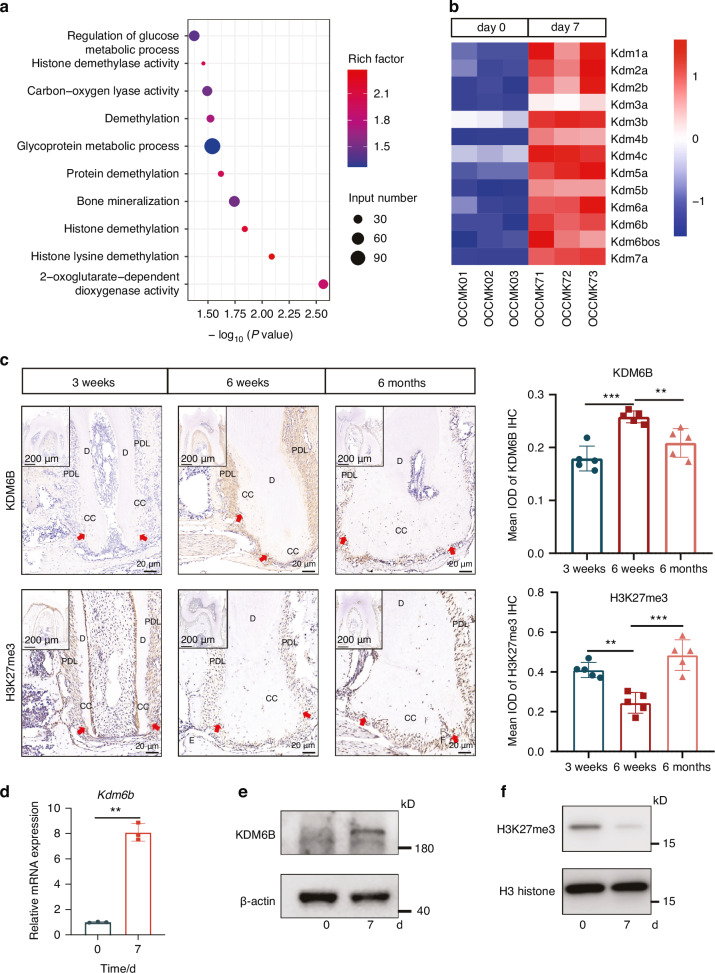
**Fig. 1** KDM6B is upregulated during cementogenesis and cementoblast mineralization. **a** Gene Ontology (GO) enrichment analysis during the cementoblast mineralization process. **b** Heatmap of histone lysine demethylases (KDMs) gene expression during cementoblast mineralization process. **c** Immunohistochemical (IHC) analysis of lysine demethylase 6B (KDM6B) and trimethylated histone H3 at lysine 27 (H3K27me3) expression in cementoblasts of 3-week-old, 6-week-old, and 6-month-old mice (*n* = 5). Red arrows indicate cementoblasts. CC cellular cementum, PDL periodontal ligament, D dentin. **d**, **e** Quantitative polymerase chain reaction (qRT-PCR) and western blotting (WB) results of KDM6B expression levels in cementoblasts at day 0 and day 7 with cementogenic induction. (*n* = 3) **f** WB results of H3K27me3 levels during cementoblast mineralization process. Data are presented as mean ± SD. **P < 0.01. ***P < 0.001

To:

**Figure:**
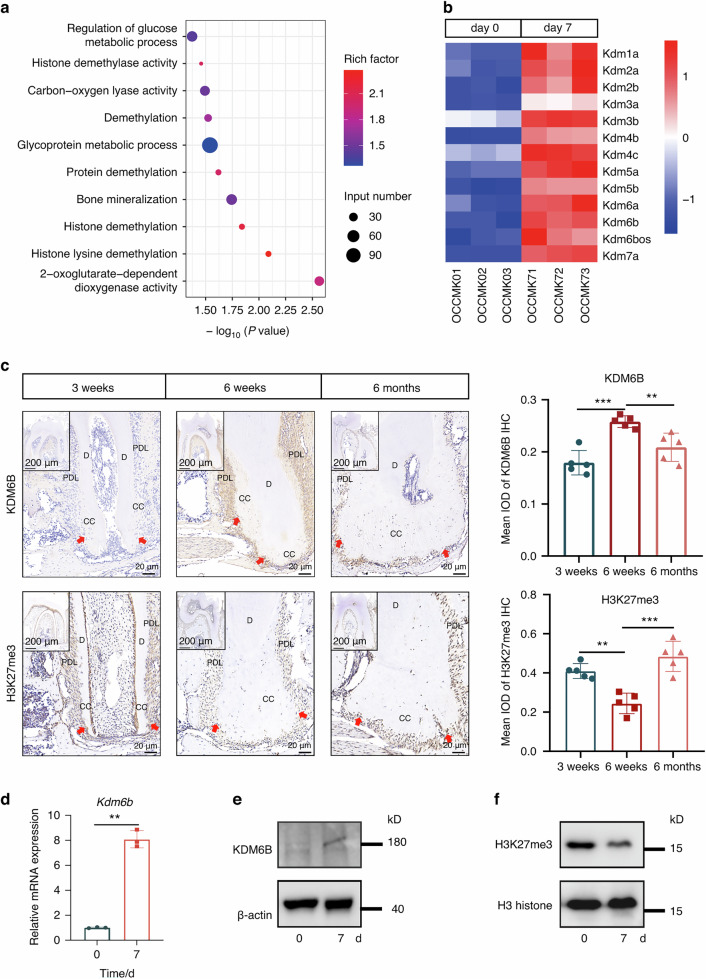
**Fig. 1** KDM6B is upregulated during cementogenesis and cementoblast mineralization. **a** Gene Ontology (GO) enrichment analysis during the cementoblast mineralization process. **b** Heatmap of histone lysine demethylases (KDMs) gene expression during cementoblast mineralization process. **c** Immunohistochemical (IHC) analysis of lysine demethylase 6B (KDM6B) and trimethylated histone H3 at lysine 27 (H3K27me3) expression in cementoblasts of 3-week-old, 6-week-old, and 6-month-old mice (*n* = 5). Red arrows indicate cementoblasts. CC cellular cementum, PDL periodontal ligament, D dentin. **d**, **e** Quantitative polymerase chain reaction (qRT-PCR) and western blotting (WB) results of KDM6B expression levels in cementoblasts at day 0 and day 7 with cementogenic induction. (*n* = 3) **f** WB results of H3K27me3 levels during cementoblast mineralization process. Data are presented as mean ± SD. **P < 0.01. ***P < 0.001

Figure 5 has been corrected from:

**Figure:**
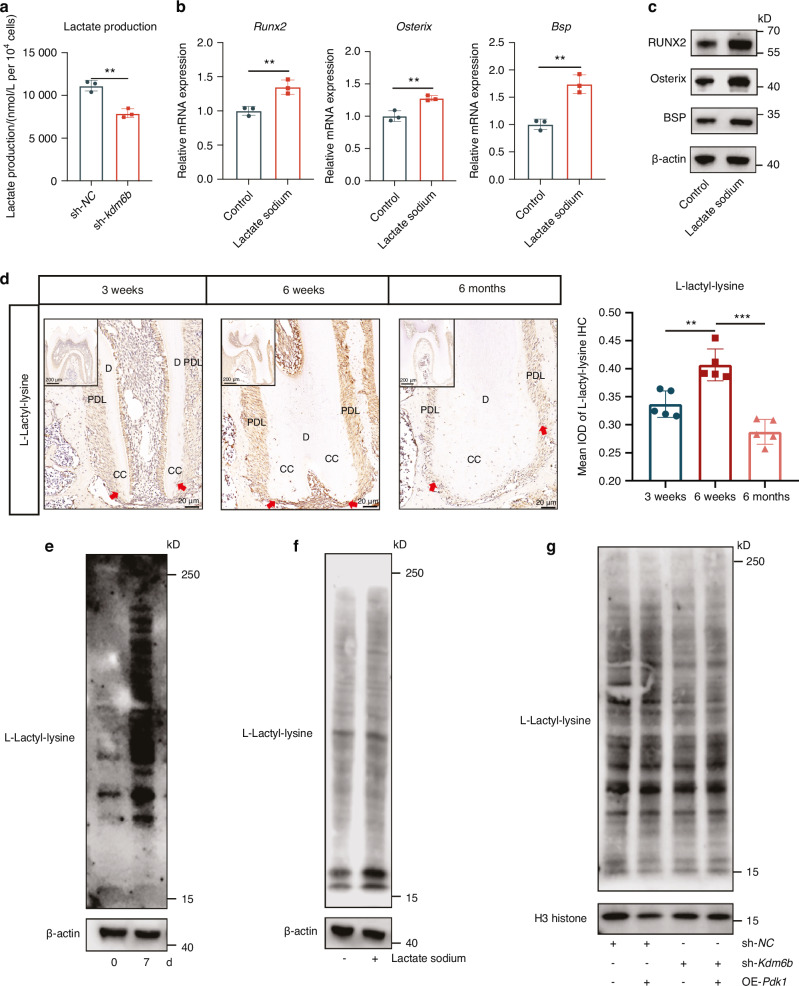
**Fig. 5** KDM6B-Pdk1-lactate axis promotes the cementoblast mineralization through lactylation. **a** Lactate production following *Kdm6b* knockdown (*n* = 3). **b**, **c** QRT-PCR and WB results for the control and lactate sodium-treated groups. **d** Levels of L-lactyl-lysine in cementoblasts at different growth stages in mice (*n* = 5). Red arrows indicate cementoblasts. CC cellular cementum, PDL periodontal ligament, D dentin. **e** Lactylation levels in cementoblasts during mineralization. **f** Lactate sodium addition increases cellular lactylation levels. **g** WB results of mineralization levels following *Pdk1* overexpression under *Kdm6b* inhibition. Data are presented as mean ± SD. **P < 0.01. ***P < 0.001

To:

**Figure:**
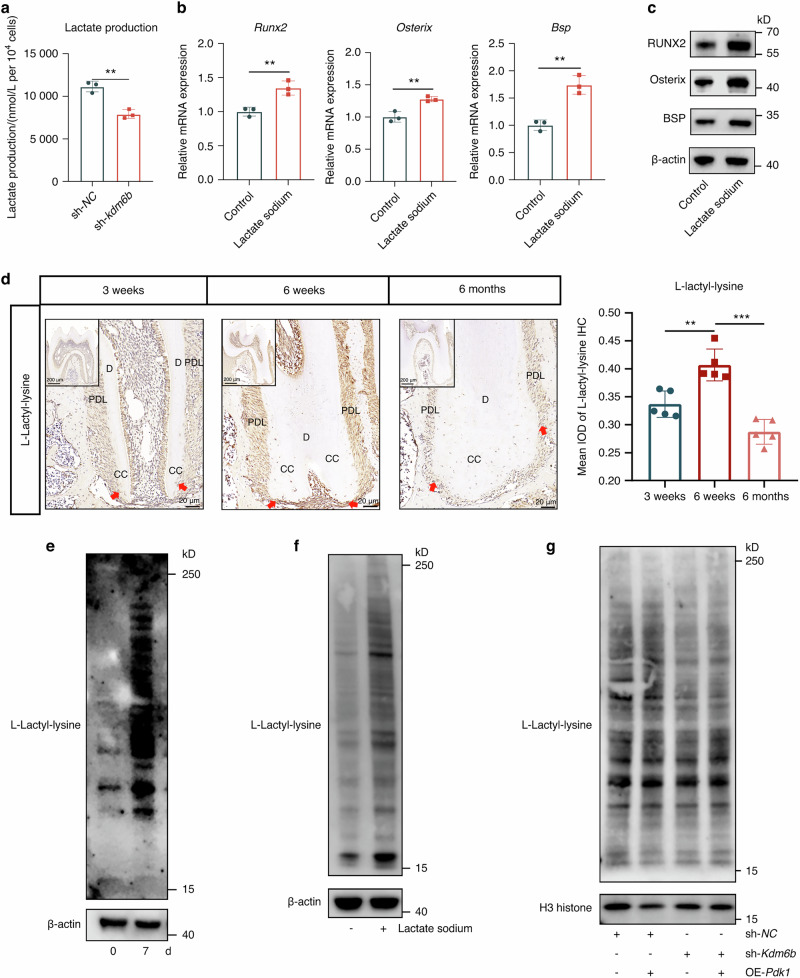
**Fig. 5** KDM6B-Pdk1-lactate axis promotes the cementoblast mineralization through lactylation. **a** Lactate production following *Kdm6b* knockdown (*n* = 3). **b**, **c** QRT-PCR and WB results for the control and lactate sodium-treated groups. **d** Levels of L-lactyl-lysine in cementoblasts at different growth stages in mice (*n* = 5). Red arrows indicate cementoblasts. CC cellular cementum, PDL periodontal ligament, D dentin. **e** Lactylation levels in cementoblasts during mineralization. **f** Lactate sodium addition increases cellular lactylation levels. **g** WB results of mineralization levels following *Pdk1* overexpression under *Kdm6b* inhibition. Data are presented as mean ± SD. **P < 0.01. ***P < 0.001

The original article^1^ has been corrected.
